# Sperm Gone Smart: A Portable Device (iSperm^®^) to Assess Semen Concentration and Motility in Dogs

**DOI:** 10.3390/ani12050652

**Published:** 2022-03-04

**Authors:** Guillaume Domain, Penelope Banchi, Hiba Ali Hassan, Anouk Eilers, Joke Lannoo, Eline Wydooghe, Wojciech Niżański, Ann Van Soom

**Affiliations:** 1 Department of Internal Medicine, Reproduction and Population Medicine, Faculty of Veterinary Medicine, Ghent University, Salisburylaan 133, 9820 Merelbeke, Belgium; penelope.banchi@ugent.be (P.B.); hiba.alihassan@ugent.be (H.A.H.); anouk.eilers@ugent.be (A.E.); joke.lannoo@ugent.be (J.L.); eline.wydooghe@ugent.be (E.W.); ann.vansoom@ugent.be (A.V.S.); 2 Department of Veterinary Sciences, University of Turin, Largo Paolo Braccini 2-5, 10095 Grugliasco, Italy; 3 Department of Reproduction and Clinic of Farm Animals, University of Environmental Science, Grundwaldzki Square 49, 50-357 Wroclaw, Poland; wojciech.nizanski@upwr.edu.pl

**Keywords:** canine, semen analysis, automated device

## Abstract

**Simple Summary:**

Semen analysis can be subjective and time-consuming if automated instruments are not available. However, such devices are expensive and not transportable for on-field analyses. A portable device (iSperm^®^) is available for the evaluation of semen concentration and motility, but data on its reliability for canine semen analysis are still scarce. This study assessed the performances of the iSperm^®^ on a large sample size (*n* = 224) by evaluating its correlation with a conventional computer-assisted sperm analyzer (ISAS^®^v1) for semen concentration and motility. The intra-assay variability of both the iSperm^®^ and the ISAS^®^v1 and their ability to estimate semen concentration at a fixed value of 40 × 10^6^/mL were also investigated. Results showed that the intra-assay variability was lower for the ISAS^®^v1 compared to the iSperm^®^. Hence, iSperm^®^ results were more variable in-between fields. Both the iSperm^®^ and the ISAS^®^v1 were not reliable in estimating semen concentration. Finally, the two devices were positively correlated, although providing different values for each parameter. Some improvements of the iSperm^®^ software are therefore needed to make it a valid alternative to automated computerized systems for the analysis of canine semen.

**Abstract:**

The iSperm^®^ is a portable device for semen analysis. This study aimed to investigate its correlation with a conventional computer-assisted sperm analyzer (ISAS^®^v1) for the assessment of semen concentration and kinematic parameters in dogs (*n* = 224). The intra-assay variability of both devices and their ability to estimate semen concentration at a fixed value of 40 × 10^6^/mL were also investigated. Results showed that the intra-assay variability was lower for the ISAS^®^v1 for all parameters compared to the iSperm^®^. Hence, iSperm^®^ estimates were more variable in-between fields. Both the iSperm^®^ and the ISAS^®^v1 were not reliable in estimating semen concentration (ISAS^®^v1: median 30 × 10^6^/mL, interquartile range (IQR) 12, *p* < 0.01; iSperm^®^: median 35.12 × 10^6^/mL, IQR 11.11, *p* < 0.01). Finally, positive correlations were found between both devices with stronger correlations obtained when four fields were analyzed by the iSperm^®^. However, the low number of spermatozoa analyzed per field and the inability to avoid artifacts are downsides that currently limit the reliability of the iSperm^®^. Therefore, the software of iSperm^®^ needs some improvement to make it a valid and practical alternative to automated computerized systems for the analysis of canine semen.

## 1. Introduction

Semen analysis is a key part of a complete breeding soundness examination in male dogs. This practice consists of both a macroscopic and microscopic evaluation of the ejaculate and is important when investigating conditions such as subfertility and infertility [1]. Specifically, the assessment of concentration, motility, and morphology allows for an estimation of the semen quality, which is related to success in both natural conception and artificial insemination [2]. The evaluation of semen motility can be performed subjectively under light microscope, but it requires training and is inherently subject to observer bias [3]. Semen concentration can be determined manually using a hemocytometer or by spectrophotometry. However, hemocytometers are time-consuming and require adequate training [4], and spectrophotometers are not able to discriminate between spermatozoa and other cells, particles, or debris that may be present in the ejaculate [5]. For this reason, more advanced and sophisticated equipment, such as computer-assisted semen analyzers (CASA) and the NucleoCounter^®^ (Chemometec, Denmark), has been developed to objectively evaluate semen motility and concentration [6,7,8]. The reliability and technical settings of CASA systems have already been extensively investigated and optimized [9,10,11,12]. The NucleoCounter^®^, on the other hand, is a newer device that has rapidly gained in popularity for the assessment of sperm concentration of several species and is now considered as the gold standard in stallions [13,14]. The NucleoCounter^®^ can discern sperm debris and particles from spermatozoa using a DNA-specific fluorescent dye that stains spermatozoa DNA. However, these automated devices are expensive and not suited for transportation, which limits their use in research and clinical facilities specifically involved in animal reproduction.

Portable, objective, and affordable devices have recently been developed to analyze semen on smartphones and tablets [15,16]. Among them, the software iSperm^®^, developed by Aidmics Biotechnology (Taipei City, Taiwan), uses the camera of an iPad Mini 4 (Apple Inc., Cupertino, CA, USA) and a heating chamber to capture and analyze multiple videoframes of semen at 37 °C. The iSperm^®^ software has species-specific settings and its use in stallions [14,17], boars [18], and stud dogs [19] has been reported in the literature. This portable tool was found accurate in estimating different semen concentrations when compared with both NucleoCounter^®^ and hemocytometer in stallions [14,17]. Results for motility assessment were encouraging in stallions and dogs [14,17,19], although accuracy in estimating velocity parameters should be improved in stallions [14]. In dogs, the strong positive correlation described between CASA systems and the iSperm^®^ for both total and progressive motility needs yet to be confirmed on a larger set of samples [19]. Moreover, the ability of the iSperm^®^ in estimating other velocity parameters is still to be investigated.

The aim of the present study was to evaluate the correlation and accuracy of the iSperm^®^ in estimating semen motility and concentration of canine spermatozoa by comparing it with a conventional CASA system (ISAS^®^v1 CASA, Proiser R + D, Paterna, Spain). The intra-assay variability of both instruments was also investigated and different combination of iSperm^®^ videoframes were tested to find the best correlation between the two instruments.

## 2. Materials and Methods

### 2.1. Animals

Stud dogs of at least one year of age presented at the teaching hospital of Ghent University for semen collection and evaluation between May 2020 and December 2021 were enrolled in the study. Exclusion criteria included hematospermia, semen concentration <40 × 10^6^ spermatozoa/mL, secretory or excretory azoospermia, and unsuccessful semen collection. Frozen-thawed samples originated from dogs presented for commercial semen cryopreservation and analyzed during routine post-thaw analysis. The final dataset of the study consisted of 136 fresh and 88 frozen-thawed semen samples, leading to 224 samples collected from 197 stud dogs, aged 48 ± 5 months (mean ± standard deviation). Animals belonged to different size categories according to Wallis et al. [20] based on their weight. Specifically, small (*n* = 7), medium-small (*n* = 27), medium-large (*n* = 60), large (*n* = 74), and giant (*n* = 29) dogs were included.

### 2.2. Semen Collection, Processing and Analysis

The sperm-rich fraction of each ejaculate was collected by digital manipulation into plastic vials as described by Linde-Forsberg [21]. After collection, the fresh semen was placed in an incubator at 37 °C and immediately analyzed. Frozen-thawed (FT) samples were obtained after immersing straws in a 37 °C water bath for 30 s [22]. Straws were then dried, and the semen was placed into warmed Eppendorf tubes. Evaluation of the FT samples was performed after 5 min incubation at 37 °C [23].

For both fresh and FT samples, semen concentration was measured using the Nucleocounter-SP100^®^ (ChemoMetec, A/S, Allerød, Denmark), according to the manufacturer’s instructions [24]. Briefly, a 10 µL aliquot of semen was diluted with 1 mL lysis reagent S100 (ChemoMetec, A/S, Allerød, Denmark) and, after mixing, was loaded into a cassette containing propidium iodide. The cassette was then inserted into the fluorescence detector of the machine and the semen concentration of the sample was reported. For an accurate assessment of motility with iSperm^®^ [19] and ISAS^®^v1 [25], semen was then diluted to a working concentration of 40 × 10^6^ spermatozoa/mL into warm and sterile saline solution (NaCl 0.9%) [6]. 

Concentration and seven kinematic parameters of all samples were investigated concurrently in both devices: total motility (TM, %), progressive motility (PM, %), average path velocity (VAP, µm/s), straight line velocity (VSL, µm/s), curvilinear velocity (VCL, µm/s), straightness (STR, %), and linearity (LIN, %).

#### 2.2.1. ISAS^®^v1

The ISAS^®^v1 (Proiser, Valencia, Spain) equipped with a heated stage set at 37 °C and a 10× negative phase-contrast objective was used as CASA system. For each field analyzed, thirty consecutive and digitized images were captured by a video digital camera (Proiser 782C, Proiser R + D, Paterna, Spain) at a frame rate of 60 fps. Four different fields containing around 200 spermatozoa per field were analyzed for each sample and the average was determined for all parameters. Tail detection was activated to allow non-sperm particles to be ignored and particle area was set between 12 and 80 µm^2^. A spermatozoon was considered immotile when presenting a VAP < 10 µm/s and spermatozoa deviating <50% from a straight line were designated as progressive. Pre-warmed ISAS^®^D4C20 disposable counting chambers (Proiser, Valencia, Spain) loaded with a 4 µL droplet of diluted semen were used to analyze fresh samples. As for FT samples, a 10 µL aliquot was mounted on a pre-warmed slide and covered by a 22 × 22 mm coverslip. The different slides used to analyze fresh and FT samples resulted from the significant drop in motility observed when ISAS^®^D4C20 disposable counting chambers were used for FT samples in comparison to subjective motility. This drop is known to be caused by the capillary action of the counting chamber [25].

#### 2.2.2. iSperm^®^

The iSperm^®^ was set up according to the guidelines of the instruction manual for canine semen. A spermatozoon was considered immotile when presenting a VAP < 15 µm/s and progressively motile when presenting a VAP ≥ 45 µm/s and deviating <30% from a straight line. For each analysis, 7.5 μL of diluted semen was placed onto the top of a pre-warmed iSperm^®^ base chip (GenePro, Fitchburg, WI, USA) and locked with a pre-warmed iSperm^®^ cover chip, as per the instruction manual. The locked chips were then immediately screwed into the heating chamber attached to the iPad Mini camera and analyzed. For each sample, four different fields containing around 50 spermatozoa per field were captured and analyzed after rotation of the locked chip (0°, 90°, 180° and 270°).

### 2.3. Statistical Analysis

Statistical analyses were performed using R version 4.1.2, 2021 (R Inc., Boston, MA, USA). The normality of the distributions was verified using histograms and Shapiro–Wilk tests and was found to be not normally distributed. The variability between the four fields captured by both ISAS^®^v1 and iSperm^®^ was estimated by the intra-assay coefficients of variation (CV) and compared using the Mann–Whitney U test. Wilcoxon signed rank test was used to assess the accuracy of the ISAS^®^v1 and the iSperm^®^ to estimate semen concentration when a fixed concentration of 40 × 10^6^/mL was used. Determination of the Spearman’s correlation coefficients (ρ) were used to assess the relationship between ISAS^®^v1 and iSperm^®^ in the estimation of semen concentration, motility (TM, PM), and other kinematic parameters (VCL, VAP, VSL, STR, and LIN). Specifically, the average value of all semen parameters obtained by the ISAS^®^v1 was compared to an increasing number of fields captured and analyzed by the iSperm^®^ (one field, two fields, three fields, or four fields), as results between each field were already found to be repeatable with the ISAS^®^v1 [7]. Correlations were considered as follows: less than 0.2 negligible association, 0.2 to 0.29 weak association, 0.3 to 0.39 moderate association, 0.4 to 0.69 strong association, and greater than 0.7 very strong association [26]. Finally, simple linear regressions were used to model the relationship between the two devices in the estimation of TM and PM, considered the most interesting parameter when evaluating semen motility [27,28]. The level of significance was set at *p* < 0.05 for all analyses.

## 3. Results

### 3.1. Intra-Assay Variability

Results for intra-assay variability (median and IQR) of all parameters are reported in Table 1. Intra-assay variability of the ISAS^®^v1 was significantly lower than the one of the iSperm^®^ for all parameters (*p* < 0.05), except for STR (*p* > 0.05). Median results of the ISAS^®^v1 were always <10%, except for concentration (12.58%, IQR 9.37%). Median results of the iSperm^®^, on the other hand, were only <10% for VCL, VAP, STR, and LIN.

### 3.2. Assessment of Semen Concentration

**Figure 1:**
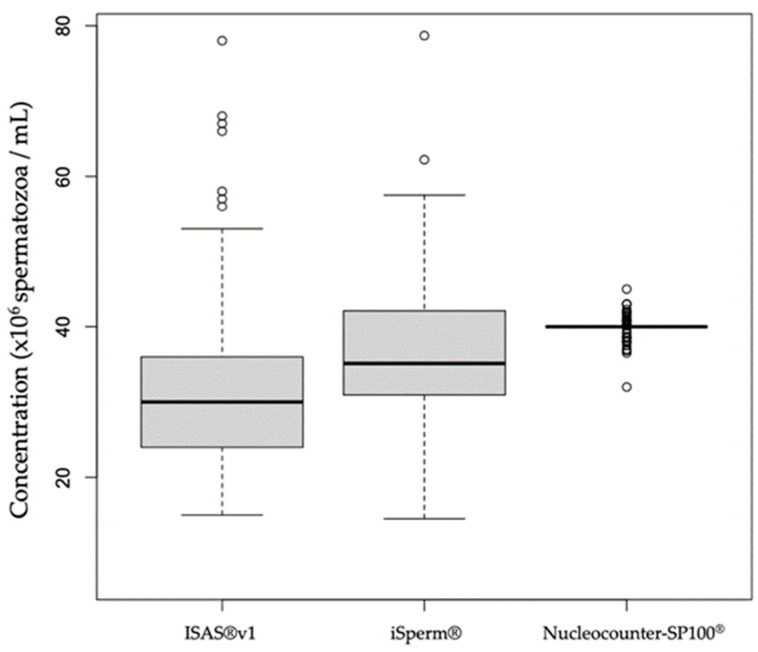
Box plots representing semen concentration measurements by ISAS^®^v1 and iSperm^®^in comparison to the set concentration of 40 × 10^6^/mL. The small circles represent outlier values.

Both ISAS^®^v1 (median: 30 × 10^6^/mL, IQR: 12) and iSperm^®^ (median: 35.12 × 10^6^/mL, IQR: 11.11) estimates of semen concentration were significantly different from the set value of 40 × 10^6^/mL (Figure 1).

### 3.3. Correlation between ISAS^®^v1 and iSperm^®^

#### 3.3.1. Concentration

**Figure 2:**
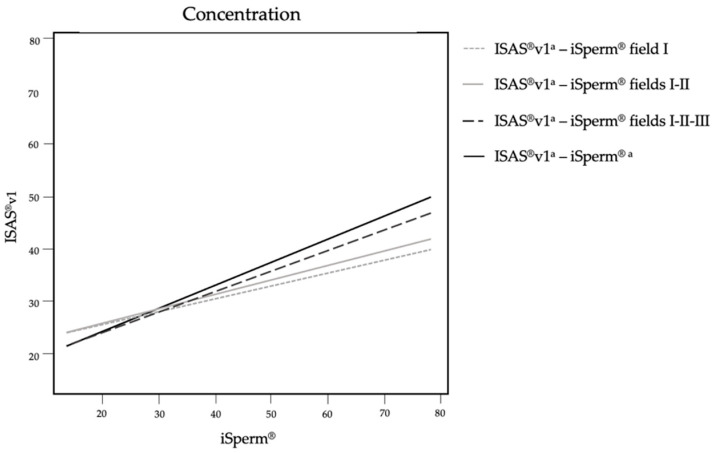
Correlation trends concerning the estimation of semen concentration on ISAS^®^v1 and iSperm^®^. The four lines resulted from the comparison of the average of the four fields analyzed by ISAS^®^v1 and different number of fields analyzed by iSperm^®^ (one, two, three or four fields). ^a^ Average of four frames.

A positive correlation was found between the ISAS^®^v1 and the iSperm^®^ for the assessment of semen concentration. The correlation was stronger, although moderate [26], when more fields were captured on the iSperm^®^ (Figure 2).

#### 3.3.2. Total and Progressive Motility

**Figure 3:**
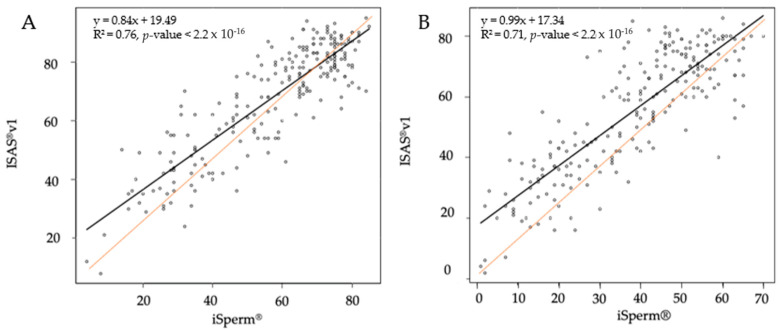
Linear regressions for total motility (**A**) and progressive motility (**B**) between the ISAS^®^v1 and the iSperm^®^ based on the average of four fields captured by both devices.

Very strong positive correlations (ρ > 0.77, *p* < 0.0001) were found between the ISAS^®^v1 and the iSperm^®^ for the estimation of total and progressive motility (data not shown). These results were independent of the number of fields analyzed and were confirmed by linear regressions (Figure 3).

#### 3.3.3. Other Kinematic Parameters

Moderate to strong positive correlations between the ISAS^®^v1 and the iSperm^®^ were found for all kinematics parameters, as shown in Table 2. Correlations between the two instruments were similar for VCL, VAP, and VSL, regardless of the number of fields analyzed by the iSperm^®^. Specifically, a strong correlation was observed for VCL, and very strong correlations were found for VAP and VSL. However, correlations were stronger for STR and LIN when more fields of the iSperm^®^ were captured and analyzed.

## 4. Discussion

The present study provides evidence that semen parameters reported by the portable device iSperm^®^ are correlated to the ones reported by a conventional CASA system (ISAS^®^v1). However, strengths of correlation vary upon parameters and increase for some parameters when more fields are captured by the iSperm^®^. Interestingly, the higher intra-assay CV of the iSperm^®^ indicates that the repeatability of this device is lower than the one of the ISAS^®^v1.

The high intra-assay variability of the iSperm^®^, already described in a previous study [19], could be explained by the limited number of spermatozoa captured and analyzed in one field (±50 spermatozoa for a concentration of 40 × 10^6^/mL). At the same concentration, the ISAS^®^v1 analyzed ±200 spermatozoa per field which limits variation between fields to less than 10% for all the parameters, except for concentration. For this reason, it should be recommended to analyze several fields when using the iSperm^®^, to allow a greater number of spermatozoa to be analyzed. Moreover, stronger correlations between the iSperm^®^ and the ISAS^®^v1 were found for concentration, STR, and LIN when four fields were analyzed in comparison to one, two, or three fields. However, the number of spermatozoa analyzed by the iSperm^®^ remained below 500, considered as the minimal acceptable number to keep the variance of estimates low, even when four fields were analyzed [3,4,11,29]. To overcome this drawback, the area of analysis of the iSperm^®^ could be increased by the manufacturer as it currently only analyzes a limited part of a field.

In this study, a positive and moderate correlation for the estimation of semen concentration was found between the ISAS^®^v1 and the iSperm^®^ when four fields were analyzed on both devices. However, these estimates were significantly different from the fixed value of 40 × 10^6^ spermatozoa/mL indicating that these devices are not reliable for estimating semen concentration, as previously described [19,30,31,32]. This lack of reliability is still under debate as other authors reported a reliable assessment of semen concentration for both devices [4,14,17].

The assessment of semen motility and kinematic parameters by both devices provided different but strongly correlated results. Progressive motility, considered as an important parameter in semen quality assessment [28], was underestimated when assessed by the iSperm^®^ in comparison to the ISAS^®^v1. As a value of PM < 70% is considered suboptimal in dogs [27,28], values obtained with the iSperm^®^ should be recalculated with the help of the linear regression equation to avoid erroneous consideration of an ejaculate as suboptimal. A value of 53.5% for PM on the portable device corresponds to 70% on the conventional CASA system and should therefore be taken as threshold value when assessing semen motility under the same settings as this study. This difference in estimates partly results from the distinct definition of progressivity and computational power between the two devices [11,12]. The reduced computational power of the iSperm^®^ affects sperm detection and assessment of velocity parameters, which in turn influence the estimation of PM [33].

Besides the iSperm^®^, other portable devices have been validated for the analysis of human and domestic animal samples. Smartphone-based semen analyzers, for instance, showed strong correlations with computerized systems and microscopic assessment, even when used by non-professionals for at-home assessment [34,35]. Similarly, the Ongo Sperm Test^®^ (Microfluidlabs, Budapest, Hungary) was found a good alternative to conventional CASA system in stallions and boars [16,36]. The development and improvement of these portable devices could therefore offer the possibility of objective semen analysis for on-field analysis and in practices where a conventional system is not affordable. However, these new devices suffer from weaknesses. As for the iSperm^®^, the area of analysis is limited and can barely be modified. Hence, a low number of spermatozoa is analyzed per field, and the possibility to avoid artifacts (e.g., air bubbles or debris) is greatly diminished. A conventional CASA system, on the other hand, allows the user to select and capture adequate fields on the slide. Moreover, the iSperm^®^ currently does not offer the opportunity to set the minimum particle area to consider an object as a spermatozoon. As a result, many small particles are wrongly considered as spermatozoa and play-back function is not available to manually delete these undesirable particles. Finally, the iSperm^®^ loses its ability to estimate semen motility as well as concentration when an ejaculate is not sufficiently diluted. In that case, no values are displayed. The practicality of this device is therefore reduced for on-field analysis as semen concentration must be assessed beforehand.

## 5. Conclusions

The iSperm^®^ is an affordable and portable device that allows for objective semen evaluation in dogs. The advantage of such a device lies primarily in the possibility to analyze semen on-field and in practices where no conventional semen analyzer is available. This study showed that results given by the iSperm^®^ are correlated with a conventional CASA system (ISAS^®^v1), but four different fields should be analyzed to improve the reliability of these estimates. The low number of spermatozoa analyzed per field, the inability to avoid artifacts, the large amount of small particles mistaken for spermatozoa, and the inaccuracy to estimate semen concentration are downsides that currently limit the reliability and practicality of this device. An improvement of the software is therefore needed to make it a valid alternative to automated computerized systems for the analysis of canine semen.

## Figures and Tables

**Table 1:** Differences in intra-assay variability results (median and interquartile range–IQR) between ISAS^®^v1 and iSperm^®^.

	ISAS^®^v1		iSperm^®^		*p*-Value
	Median %	IQR %	Median %	IQR %	
Concentration	12.58	9.37	14.69	9.05	0.006 *
Total motility (%)	7.05	9.30	12.53	11.51	2.09 × 10^−11^ *
Progressive motility (%)	8.11	10.19	17.07	18.75	5.61 × 10^−19^ *
Curvilinear velocity (µm/s)	3.45	2.81	8.12	5.31	7.85 × 10^−32^ *
Average path velocity (µm/s)	5.02	4.20	9.00	5.74	9.58 × 10^−20^ *
Straight line velocity (µm/s)	7.26	5.92	10.44	6.93	2.37 × 10^−10^ *
Straightness (%)	3.14	3.30	3.00	2.28	0.37
Linearity (%)	5.82	5.40	4.11	3.30	1.8 × 10^−9^ *

* Significance for *p* < 0.05.

**Table 2:** Spearman correlation coefficients (ρ) between the ISAS^®^v1 and different number of fields analyzed by the iSperm^®^ for some kinematic parameters: curvilinear linear velocity (VCL), average path velocity (VAP), straight line velocity (VSL), straightness (STR), and linearity (LIN).

	VCL		VAP		VSL		STR		LIN	
	ρ	*p*-Value	ρ	*p*-Value	ρ	*p*-Value	ρ	*p*-Value	ρ	*p*-Value
ISAS^®^v1 ^a^–iSperm^®^ field I	0.60	1.19 × 10^−23^ *	0.76	1.62 × 10^−43^ *	0.76	1.39 × 10^−4^ *	0.39	1.71 × 10^−09^ *	0.64	4.33 × 10^−27^ *
ISAS^®^v1 ^a^–iSperm^®^ fields I-II	0.59	9.08 × 10^−23^ *	0.77	2.16 × 10^−46^ *	0.79	8.94 × 10^−49^ *	0.46	4.27 × 10^−13^ *	0.67	2.54 × 10^−31^ *
ISAS^®^v1 ^a^–iSperm^®^ fields I-II-III	0.60	1.83 × 10^−23^ *	0.77	5.31 × 10^−46^ *	0.78	1.04 × 10^−47^ *	0.50	1.32 × 10^−15^ *	0.67	1.07 × 10^−30^ *
ISAS^®^v1 ^a^–iSperm^® a^	0.61	2.20 × 10^−18^ *	0.77	2.98 × 10^−46^ *	0.79	4.02 × 10^−50^ *	0.52	3.32 × 10^−17^ *	0.70	3.93 × 10^−34^ *

^a^ Average of four frames; * Significance for *p* < 0.05.

## Data Availability

The data presented in this study are available on request from the corresponding author.
